# A clinicopathological and genetic study of sporadic diffuse leukoencephalopathy with spheroids: a report of two cases

**DOI:** 10.1111/nan.12046

**Published:** 2013-10-29

**Authors:** T Kimura, K Ishizawa, T Mitsufuji, T Abe, Y Nakazato, K Yoshida, A Sasaki, N Araki

**Affiliations:** * Department of Neurology, Saitama Medical UniversityMoroyama-town, Japan; † Department of Pathology, Saitama Medical UniversityMoroyama-town, Japan; ‡ Department of Medicine (Neurology and Rheumatology), Shinshu University School of MedicineMatsumoto, Japan

Diffuse leukoencephalopathy with spheroids (DLS) is a white matter neurodegenerative disease characterized by progressive cognitive decline and motor symptoms 1–6, and histologically, by axonal swellings (‘spheroids’) and loss of axons and myelin 1–3,5,7–11. It was originally described as a rare, hereditary, autosomal dominant disorder (hereditary DLS: HDLS) 2, but there have been reports on DLS without family history as well (sporadic DLS: SDLS) 6,12–22. In 2012, Rademakers *et al*. 9 identified 14 different mutations in the colony stimulating factor 1 receptor (*CSF1R*) gene, which are located in exons 12–22 and affect the tyrosine kinase domain of the protein, in 14 families with HDLS. Interestingly, this gene shares the same signalling pathway as *TYROBP (DAP12)* and *TREM2*, whose mutations are implicated in polycystic lipomembranous osteodysplasia with sclerosing leukoencephalopathy (PLOSL, also known as Nasu-Hakola disease) 23–25. PLOSL shares similar clinicopathological profiles with DLS, such as a progressive neuropsychiatric decline and leukoencephalopathy with spheroids 26,27. In this paper we describe the clinicopathological features of two cases of SDLS. In one of them, genetic analyses of *CSF1R*, *TYROBP* and *TREM2* were conducted, and no mutations in these genes were identified.

**Figure 1:**
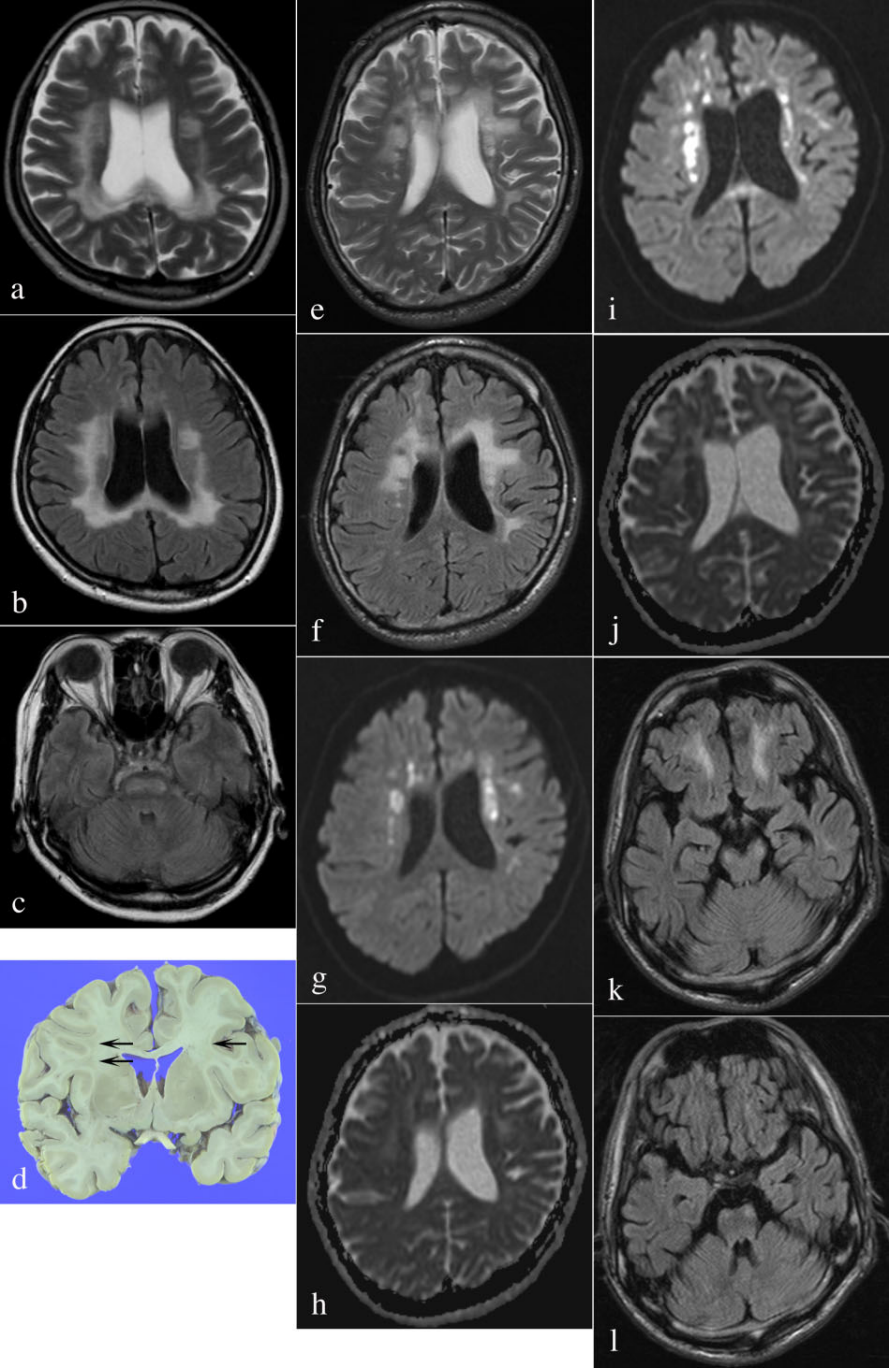
The MRI of Case 1 (a–c) shows a high-intensity signal in the cerebral white matter and corpus callosum on T2-WI (a) and FLAIR (b). This signal descends along the pyramidal tract, which is demonstrated on FLAIR in the pontine base (c). Grossly, the autopsied brain of Case 1 (d) shows bilateral, patchy discoloration of the white matter (arrows). The MRI of Case 2 (e–l) shows a high-intensity signal in the cerebral white matter on T2-WI (e) and FLAIR (f). This signal is partially high on DWI (g) and low on ADC (h), which is reminiscent of acute infarction. The follow-up MRI about 1 year after the onset shows persistence of the previously recognized signal, which is still high on DWI (i) and low on ADC (j). In addition, a previously unrecognized abnormal signal is apparent along the pyramidal tract, which is shown on FLAIR in the cerebral peduncle (k) and pontine base (l). MRI, magnetic resonance imaging; T2-WI, T2-weighted image; FLAIR, fluid attenuated inversion recovery; DWI, diffusion weighted image; ADC, apparent diffusion coefficient map.

Case 1, a 56-year-old woman presented with forgetfulness and an inability to count. She was hospitalized 8 months after the onset. Her past history included cured uterine cervical cancer at the age of 25. There was no history of bone fractures. She had no relatives with neurological diseases. At presentation, she scored a 24/30 on the mini-mental state examination, and showed left-sided hemispatial agnosia, apraxia, and ‘alien hand syndrome’ in the left hand. Frontal release signs were positive. There was no weakness in the limbs. Jaw reflex was exaggerated, as were deep tendon reflexes. Extensor plantar reflex was bilaterally positive. The sensation on the left side of the body was ignored when both sides of the body were simultaneously stimulated. Cerebellar functions were normal. Routine haematology was normal. Other tests were noncontributory, including lactate, pyruvate, vitamins, thyroid, Treponema pallidum haemagglutination test, anti-human immunodeficiency virus antibodies, very long chain fatty acid, galactocerebrosidase, arylsulphatase A, and point mutations of the genes for glial fibrillary acidic protein and *notch 3*. Anti-nuclear antibodies (ANA, centromere type), but not other autoantibodies, were elevated (>×1280). Cerebrospinal fluid (CSF) was unremarkable, including IgG index, oligoclonal band, polymerase chain reaction (PCR) for JC virus, tau, and 14-3-3 protein. Gadolinium-enhanced magnetic resonance imaging (MRI) disclosed a non-enhanced, high-intensity signal in the cerebral white matter on T2-weighted (Figure 1**a**) and fluid attenuated inversion recovery (FLAIR) (Figure 1**b**) images, which descended along the pyramidal tract (Figure 1**c**). This signal was high on diffusion weighted image (DWI) and apparent diffusion coefficient (ADC) map. Given the elevated ANA, immunomodulating therapy was attempted, but there was no effect. She gradually deteriorated and eventually became bed-ridden. She died 16 months after the onset. Autopsy limited to the brain was performed.

Case 2, a 35-year-old man presented with mutism. His family gradually noticed that he would not utter or talk. He then began to limp and was hospitalized 3 months after the onset. His past history was unremarkable, with no episodes of bone fractures. One of his uncles and one of his grandmothers died of cerebral haemorrhage; otherwise, his family history was unremarkable. At presentation, he uttered no words and failed to follow commands. There was no weakness in the limbs. Deep tendon reflexes were exaggerated, and extensor plantar reflex was positive on the right side. Sensation and cerebellar functions seemed normal. Gadolinium-enhanced MRI disclosed a non-enhanced, high-intensity signal in the cerebral white matter on T2-weighted (Figure 1**e**) and FLAIR (Figure 1**f**) images, which was high on DWI (Figure 1**g**) and low on ADC (Figure 1**h**). The pyramidal tract seemed spared. Routine haematology was normal. Very long chain fatty acid, arylsulphatase A, and *notch 3* gene were normal. In CSF, protein (50 mg/dl) and myelin basic protein (134 pg/ml) were elevated; otherwise, the CSF was normal. Given the signal pattern on MRI, that is, high on DWI and low on ADC, a tentative diagnosis of acute infarction was made, but its aetiology remained undetermined. One year after the onset, the patient had prominent bulbar palsy that led to severe dehydration. On the follow-up MRI, the previously recognized signal pattern, that is, high on DWI and low on ADC, was still present (Figure 1**i**,**j**), and a previously unrecognized signal became obvious along the pyramidal tract (Figure 1**k**,**l**). Brain biopsy was performed 1 year and 3 months after the onset.

Histology was evaluated with haematoxylin and eosin (HE), Klüver–Barrera (KB), HE-periodic acid-Schiff stain (HE-PAS), luxol fast blue (LFB)-PAS and/or Bodian stains using formalin-fixed, paraffin-processed sections. Also, immunostains were performed using the following antibodies: SMI31 (1:10 000, Sternberger Monoclonals Incorpotated, Lutherville, MD, USA) and/or 2F11 (1:100, DakoCytomation, Carpinteria, CA, USA) for phosphorylated neurofilament (p-NF); LN27 (1:400, Zymed, South San Francisco, CA, USA) for amyloid beta-precursor protein (APP); 4G8 (1:20 000, Senetek, St. Louis, MO, USA) for amyloid beta; AT8 (1:2000, Innogenetics, Ghent, Belgium) for phosphorylated tau; #64 (1:10 000, Courtesy of Dr T. Iwatsubo, Tokyo University 28) for phosphorylated alpha-synuclein; an antibody for TAR-DNA-binding protein-43 (TDP-43, polyclonal; 1:100, ProteinTech Group, Chicago, IL, USA).

Genetic analyses of *CSF1R, TYROBP* and *TREM2* were conducted in Case 1, according to the methods described in previous papers 9,23–25,29. Briefly, genomic DNA was extracted using Gentra Puregene Blood Kit (Qiagen) from the frozen block of the frontal lobe, and the whole exons of each gene were amplified by polymerase chain reaction (PCR). Direct sequence analyses were applied to the PCR-amplified DNA.

**Figure 2:**
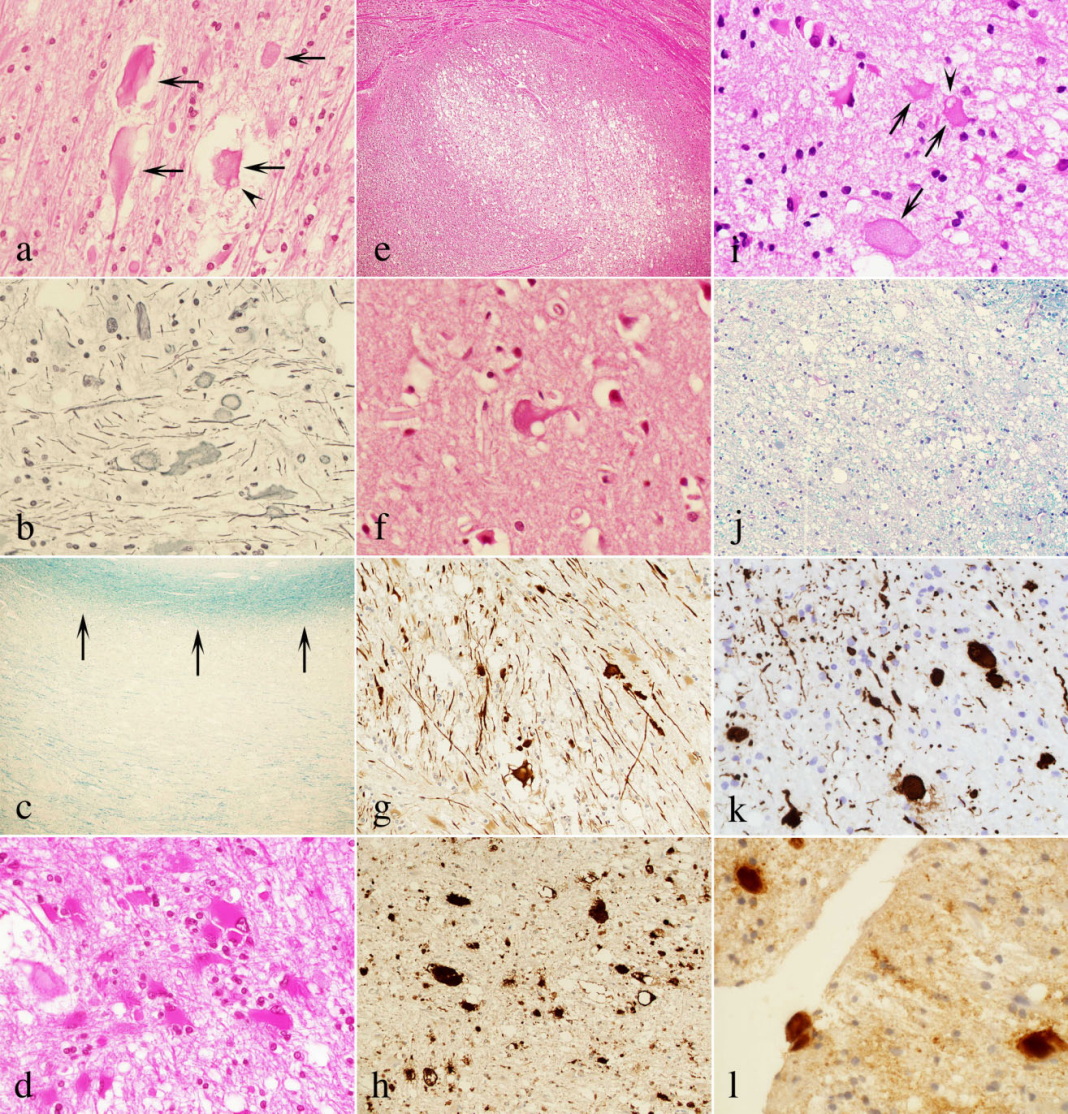
Histological and immunohistochemical findings of Case 1 (a–h) and Case 2 (i–l). *Case 1:* (a) Many spheroids are present in the white matter (arrows). Note a spheroid showing vacuolation (arrowhead). (HE, original magnification ×400.) (b) Many spheroids are clearly visualized with Bodian stain. The density of axons is markedly decreased. (Bodian stain, ×400.) (c) While the deep white matter shows marked loss of myelin, the U-fibres underlying the cortex show preservation of myelin (arrows). (KB, ×40.) (d) Clumps of bizarre reactive astrocytes are noted. (HE, ×400.) (e) The longitudinal fibres (pyramidal tract) of the pontine base are markedly rarefied and vacuolated. (HE, ×40.) (f) Ballooned neurones are sporadically noted in the cortex. The one shown here has cytoplasmic vacuolation. (HE, ×600.) (g) The spheroids are immunoreactive for p-NF. (SMI31-immunostain, ×200.) (h) The spheroids are immunoreactive for APP. (LN27-immunostain, ×200.) *Case 2:* (i) Many spheroids are present in the white matter (arrows). Note a spheroid showing vacuolation (arrowhead). In the left upper field, bizarre reactive astrocytes are aggregated. (HE, original magnification ×600.) (j) Marked loss of myelin is present in the white matter. (LFB-PAS, ×200.) (k) The spheroids are immunoreactive for p-NF. Marked loss of axons is also obvious. (2F11-immunostain, ×400.) (l) The spheroids are immunoreactive for APP. (LN27-immunostain, ×600.) HE, haematoxylin and eosin; KB, Klüver–Barrera; p-NF, phosphorylated neurofilament; LFB, luxol fast blue; PAS, periodic acid-Schiff; APP, amyloid beta-precursor protein.

In Case 1, the brain weighed 1320 g and showed discolouration and attenuation of the deep white matter, particularly in the frontal lobe (Figure 1**d**). Histology showed the following white matter changes: marked rarefaction and vacuolation; many spheroids (Figure 2**a**,**b**); marked loss of axons (Figure 2**b**) and myelin (Figure 2**c**); dysmorphism of the spheroids (Figure 2**a**); marked astrocytosis, including bizarre reactive astrocytes (Figure 2**d**); and scattered macrophages. These changes descended along the pyramidal tract (Figure 2**e**). In the cortex, ballooned neurones were noted sporadically (Figure 2**f**). The U-fibres were well preserved (Figure 2**c**). The basal ganglia and the cerebellum were unremarkable. By immunohistochemistry, the spheroids were immunoreactive for p-NF (Figure 2**g**) and APP (Figure 2**h**).

The biopsy material of Case 2 consisted of white matter. As in Case 1, the histology showed the following: many spheroids (Figure 2**i**); loss of axons (Figure 2**k**) and myelin (Figure 2**j**); dysmorphism of the spheroids (Figure 2**i**); marked astrocytosis, including bizarre reactive astrocytes (Figure 2**i**); and scattered macrophages. A few micro-calcifications were seen. By immunohistochemistry, the spheroids were immunoreactive for p-NF (Figure 2**k**) and APP (Figure 2**l**).

In both cases, there were neither vascular or neoplastic changes nor abnormal structures immunoreactive for amyloid beta, phosphorylated tau, phosphorylated alpha-synuclein or TDP-43.

The genetic analyses of *CSF1R, TYROBP* and *TREM2* in Case 1 identified no mutations all through the exons of the three genes (exons 1–22, *CSF1R*; exons 1–5, *TYROBP*; exons 1–5, *TREM2*).

The leukoencephalopathies involving axons are grouped under the umbrella term ‘neuroaxonal dystrophy (NAD)’ 27. NAD is divided into three categories: physiological NAD, secondary NAD and primary NAD 27. Physiological NAD is due to ageing, and is seen in the gracile and cuneate nuclei. The present cases are pathologically different from physiological NAD in the neuroanatomical distribution and severity of the lesions. Secondary NAD stems from various conditions, such as Parkinson's disease, motor neurone diseases or neuronal storage diseases. In the present cases, secondary NAD was excluded by the clinical work-up and the pathological assessment. Primary NAD corresponds to neurodegeneration involving axons, such as infantile neuroaxonal dystrophy, neurodegeneration with brain iron accumulation type I, or PLOSL. These are hereditary, and tend to have a more systemic manifestation and an earlier onset than do the present cases. Among these, PLOSL in particular needs to be differentiated from the present cases; however, the present cases lack bone symptoms, and in Case 1, the genetic analyses of *TYROBP* and *TREM2* excluded PLOSL. Taken together with the pathological findings, the present cases can be diagnosed as DLS, one of primary NAD.

The neuroradiology of DLS includes increased signal intensity in the subcortical white matter and the pyramidal tract on T2-weighted or FLAIR MRI 6,13,21,30 with or without gadolinium enhancement 8,30. Recently, a few studies have shown that white matter lesions of DLS can be high on DWI and low on ADC 13,15,30, potentially mimicking acute infarction. For example, Mateen *et al*. 13 reported a 24-year-old woman of SDLS with a 7-month course of dysarthria and gait impairment. Brain MRI showed T2 signal abnormality in periventricular and subcortical regions with extension into the right internal capsule and cerebral peduncle. Several areas were high on DWI and low on ADC, which led to a diagnosis of acute infarction; but this signal persisted even after 19 weeks. Brain biopsy was performed, and a diagnosis of DLS was established. This case and the present Case 2 are exactly comparable to each other, suggesting that the signal pattern on MRI in DLS can lead to a misdiagnosis of acute infarction.

The histology of HDLS features white matter degeneration including spheroids, loss of axons and myelin, and bizarre reactive astrocytes 1–3,5,7–11, which can descend along the pyramidal tract 1–5,8,10. Although the cerebral cortex is generally spared, ballooned neurones can occasionally be present 1,5,8,9,11. Subcortical U-fibres are well preserved 1–4,6,7,10. On the other hand, there have been some studies of the histology of SDLS 6,12–22. Table 1 reviews the histology of SDLS in a total of 13 studies available, including ours. As this table suggests, the histology of SDLS can faithfully replicate that of its hereditary counterpart. The striking similarity of the histology of SDLS to that of HDLS strongly suggests that SDLS may also be genetically determined, as is HDLS.

**Table:** The histology of sporadic diffuse leukoencephalopathy with spheroids

													Present	
	Goodman (1995) 17	van der Knaap (2000) 6	Yamashita (2002) 18	Browne (2003) 16	Moro-de-Casillas (2004) 21	Mascalchi (2006) 22	Mayer (2007) 14	Keegan (2008) 20	Levin (2008) 19	Maillart (2009) 15	Mateen (2010) 13	Wong (2011) 12	Case 1	Case 2
Diagnostic procedure	Ax	Ax	Ax	Ax	Ax	Bx	Bx	Bx	Bx	Bx	Bx	Ax	Ax	Bx
Age at diagnosis, sex	51, M	39, NA	51, F	54, M	67, M	41, F	42, M	39, M; 41, M; 54, F; 55, F; 42, F	57, M	33, F; 39, F	24, F	60, M	56, F	35, M
**Histology**														
Spheroids	+	+	+	+	+	+	+	+	+	+	+	+	+	+
Loss of axons and/or myelin	+	+	+	+	+	+		+	+	+	+	+	+	+
Pyramidal tract degeneration	+	+	+	+									+	
Bizarre reactive astrocytes										−			+	+
Preserved U-fibres		+	+	+						+		+	+	
Ballooned neurones		−	−	−	−	−			−	−			+	

A blank indicates that no data or descriptions are available. + , present; −, absent; M, male; F, female; NA, not available; Ax, autopsy; Bx, biopsy.

In 2012 Rademakers *et al*. 9 identified 14 different mutations of *CSF1R*, which are located in exons 12–22 affecting the tyrosine kinase domain of the protein, in 14 families with HDLS. Kinoshita *et al*. 29 identified another novel mutation of *CSF1R*, a heterozygous c.2345 G>A (p.782 Arg>His) in exon 18, in a Japanese family with HDLS. In the present study, we performed a direct sequence analysis of *CSF1R*, *TYROBP* and *TREM2* in Case 1, a case of SDLS. This case was considered as pathologically proven DLS with no apparent family history of neuropsychiatric disorders, and did not carry any mutations in the coding regions and exon-intron boundaries of *CSF1R*, *TYROBP* or *TREM2*. Therefore, this case might be a phenocopy of HDLS; however, the possibility that rare mutations in one of these genes or unidentified genes responsible for HDLS are operative in this patient cannot totally be excluded. DLS, encompassing HDLS and SDLS, needs to be taken as a disease spectrum with a heterogeneous genetic background.
